# Implications for Combination Therapy of Selective Monoamine Reuptake Inhibitors on Dopamine Transporters

**DOI:** 10.3390/biomedicines11102846

**Published:** 2023-10-20

**Authors:** Hyomin Ahn, Kichul Park, Dongyoung Kim, Sung-Gil Chi, Kee-Hyun Choi, Seo-Jung Han, Chiman Song

**Affiliations:** 1 Chemical & Biological Integrative Research Center, Korea Institute of Science and Technology, 5 Hwarang-ro 14-gil, Seongbuk-gu, Seoul 02792, Republic of Koreakeehyun.choi@gmail.com (K.-H.C.); 2 Department of Life Sciences, Korea University, 145 Anam-ro, Seongbuk-gu, Seoul 02841, Republic of Korea; chi6302@korea.ac.kr; 3 OZIWORX, R&D Laboratory, 130-2, Donghwagongdan-ro, Gangwon-do, Wonju-si 26365, Republic of Korea; 4 Division of Bio-Medical Science & Technology, KIST School, University of Science and Technology, Seoul 02792, Republic of Korea

**Keywords:** monoamine transporter, dopamine transporter, antagonistic effect, vanoxerine, nisoxetine, fluoxetine

## Abstract

Monoamine transporters, including dopamine, norepinephrine, and serotonin transporters (DAT, NET, and SERT, respectively), are important therapeutic targets due to their essential roles in the brain. To overcome the slow action of selective monoamine reuptake inhibitors, dual- or triple-acting inhibitors have been developed. Here, to examine whether combination treatments of selective reuptake inhibitors have synergistic effects, the pharmacological properties of DAT, NET, and SERT were investigated using the selective inhibitors of each transporter, which are vanoxerine, nisoxetine, and fluoxetine, respectively. Potencies were determined via fluorescence-based substrate uptake assays in the absence and presence of other inhibitors to test the multi-drug effects on individual transporters, resulting in antagonistic effects on DAT. In detail, fluoxetine resulted in a 1.6-fold increased IC_50_ value of vanoxerine for DAT, and nisoxetine produced a more drastic increase in the IC_50_ value by six folds. Furthermore, the effects of different inhibitors, specifically monovalent ions, were tested on DAT inhibition by vanoxerine. Interestingly, these ions also reduced vanoxerine potency in a similar manner. The homology models of DAT suggested a potential secondary inhibitor binding site that affects inhibition in an allosteric manner. These findings imply that the use of combination therapy with monoamine reuptake inhibitors should be approached cautiously, as antagonistic effects may occur.

## 1. Introduction

Monoamine transporters, including the dopamine (DAT), norepinephrine (NET), and serotonin transporters (SERT), are localized to presynaptic sites, where they terminate monoamine transmission signaling via reducing the synaptic concentrations of neurotransmitters and maintain presynaptic monoamine storage [1]. Neurotransmitter vesicles release neurotransmitters into the synaptic cleft. Neurotransmitters bind to specific receptors on the postsynaptic cell. Some of the remaining neurotransmitters are decomposed by monoamine oxidase and catechol-*O*-methyl transferase in the cleft, and some are reuptaken by specific transporters on the presynaptic cell. DAT, NET, and SERT are members of the solute carrier 6 (SLC6) transporter family. While SERT is highly selective to serotonin, DAT and NET are less selective; their endogenous substrates are dopamine, norepinephrine, and epinephrine. Importantly, the force driving monoamine transporters to uptake neurotransmitter is the concentration gradients of Na^+^ and Cl^−^ [2]. DAT transports two Na^+^ and one Cl^−^ with one dopamine, and NET transports one Na^+^ and one Cl^−^ with one norepinephrine. SERT transports one Na^+^ and one Cl^−^ with one serotonin and anti-transports one K^+^. The crystal structures of a bacterial leucine transporter (LeuT) in the SLC family have been determined, revealing that a substrate (leucine) binding site is located between the first and sixth transmembrane domains [3].

Monoamine transporters are important therapeutic targets [1]. DAT is involved in depression, schizophrenia, Parkinson’s disease, and attention deficit hyperactivity disorder. NET has implications in depression and pain, and SERT is related to depression, pain, anxiety, and obsessive-compulsive disorder. The pharmacology of monoamine transporters has been extensively established; thus, selective drugs for each monoamine transporter have been well developed. In recent years, triple-acting agents simultaneously inhibiting DAT, NET, and SERT have been actively developed for treating depression because they have a faster onset of action and better efficacy than current antidepressants due to their dopamine components [4]. In addition, triple-acting drugs could be developed for pain therapy as well. Given the well-established pharmacology of each transporter, we were curious whether treatment with a combination of drugs that inhibit different targets would have synergistic effects. In this work, we examined the feasibility of treatment with combinations of multiple drugs.

## 2. Materials and Methods

### 2.1. Cell Culture and Preparation

HEK293 cells stably expressing human DAT (HEK-hDAT), human NET (HEK-hNET), or human SERT (HEK-hSERT) were kindly provided by Professor Bryan Roth from the University of North Carolina at Chapel Hill. Cells were cultured in Dulbecco’s modified Eagle’s medium (Welgene, Daegu, Republic of Korea) supplemented with 10% (*v*/*v*) fetal bovine serum (Welgene, Daegu, Republic of Korea), penicillin (100 U/mL, Welgene, Daegu, Republic of Korea), and streptomycin (100 μg/mL, Welgene, Daegu, Republic of Korea) as well as selection antibiotics, geneticin G418 (350 μg/mL, 200 μg/mL, and 500 μg/mL for HEK-hDAT, HEK-hNET, and HEK-hSERT, respectively, Gibco, Frederick, MD, USA) in a humidified 5% CO_2_ incubator at 37 °C. Cells were passaged every three days. Eighteen to twenty hours prior to the cell-based uptake assay, cells were seeded onto the poly-L-lysine-coated (0.05 mg/mL, Sigma-Aldrich, St. Louis, MO, USA) 96-well black wall/clear-bottom plates (NUNC, Rochester, NY, USA) at a density of 5 × 10^4^ cells per well.

### 2.2. Neurotransmitter Uptake Assay

Dopamine, norepinephrine, and serotonin uptake activities were measured using a Neurotransmitter Transporter Uptake Assay Kit (Molecular Devices, Sunnyvale, CA, USA) [5] with an FDSS6000 plate reader, a high-throughput screening device (Hamamatsu Photonics, Hamamatsu, Japan). Cells cultured in 96-well plates were washed three times with HEPES-buffered solution (150 mM NaCl, 5 mM KCl, 10 mM glucose, 2 mM CaCl_2_, 1 mM MgCl_2_, 10 mM HEPES, at pH 7.4). Fluorescent neurotransmitter substrates were added to cells and incubated for 10 min at room temperature. The changes in intracellular substrate concentrations were monitored for 30 min as a ratio of the measured fluorescent intensity to the initial fluorescent intensity (F_t_/F_0_). In detail, cells were selectively exposed to 440 nm light for excitation, and the emitter fluorescence light through a 515 nm long-pass filter was passed with a freezing digital CCD camera mounted on the device. Data were collected every 90 s at 520 nm using a digital fluorescent analyzer. For the uptake inhibition assay, cells were pretreated with test inhibitors for 15 min in a humidified 5% CO_2_ incubator at 37 °C prior to the substrate addition. Vanoxerine (GBR12909), nisoxetine, and fluoxetine (Tocris Bioscience, Ellisville, MO, USA) were used as selective drugs for DAT, NET, and SERT, respectively. To obtain the dose–response curves, the percent transporter activity (% of control activity) was calculated using the fluorescence ratios (F_t_/F_0_) at 30 min for the inhibitor-treated and untreated cells. All data were acquired and analyzed using an FDSS6000 system and its software.

### 2.3. Computational Method

The three-dimensional structure of human DAT was obtained from the AlphaFold Protein Structure Database (https://alphafold.ebi.ac.uk/ (accessed on 13 May 2022)). AutoDock Vina (https://vina.scripps.edu/ (accessed on 13 May 2022)) was employed to predict the complex structure between hDAT and the inhibitors. Protein dynamics was simulated using the GROMACS MD simulation package 2022.1 (https://www.gromacs.org/ (accessed on 13 May 2022)).

### 2.4. Data Analysis

The dose–response curves were fitted to a Hill equation (sigmoidal dose-response equation), where %A*_min_* and %A*_max_* are the minimum and maximum percent activity of monoamine transporters, respectively. IC_50_ is the half-maximum inhibition concentration, *C* is the logarithm of concentration, and *h* is the Hill coefficient.

%A = %A*_min_* + (%A*_max_* − %A*_min_*)/(1 + 10^((LogIC50 − C) × h)^)

All results are presented as the mean ± SEM. The significance of observed differences was evaluated using Kruskal–Wallis and post hoc Dunn’s tests. A *p* value of <0.05 was considered statistically significant, where * *p* < 0.05, ** *p* < 0.01, *** *p* < 0.001, and **** *p* < 0.0001. NS is not significant.

## 3. Results and Discussion

### 3.1. Antagonistic Effects of Multiple Drugs on DAT Inhibition

**Figure 1:**
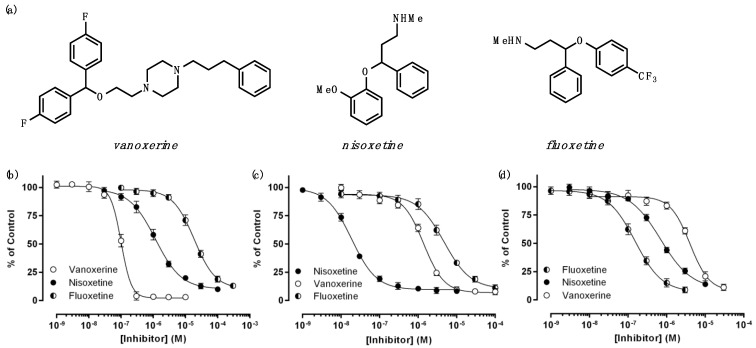
Dose–response curves of drugs against monoamine transporters. (**a**) The structures of vanoxerine, nisoxetine, and fluoxetine. Dose–response curves of vanoxerine, nisoxetine, and fluoxetine against DAT (**b**), NET (**c**), and SERT (**d**).

To investigate the effects of multiple drugs on monoamine transporter inhibition, selective drugs for each monoamine transporter were tested using HEK293 cells expressing DAT, NET, or SERT. The time-dependent uptake of neurotransmitters was observed in all of the three transporters, which is presented as a ratio of the measured fluorescent intensity to the initial fluorescent intensity (F_t_/F_0_) in Figure 1. The concentration-dependent inhibition of substrate uptake us also shown as DAT inhibition by vanoxerine, NET inhibition by nisoxetine, and SERT inhibition by fluoxetine (Figure S1) and is presented as a percent transporter activity (% of control) as a function of inhibitor concentration in Figure 1. The data from the dose–response curve fittings are summarized in Table 1.

The IC_50_ values obtained in this work match the previously reported values in the rank of drug potencies for each transporter (Figure S2 and Table 1) [6,7,8]. However, there are systematic differences in the absolute numbers of the IC_50_ values between the experimental data and the literature data; experimentally obtained values are systematically bigger than the values from the literature, with the experimental IC_50_/literature IC_50_ ratios ranging from 0.9 to 8.0 (Figure S2 and Table 1). These differences are likely due to the differences in substrate uptake assays. For example, fluorescence-labeled neurotransmitters were used for continuous measurements in the current study, while the reference values were obtained from uptake assays using radio-labeled neurotransmitters in a non-continuous format. Continuous verses non-continuous measurements as well as differently labeled substrates would lead to the different IC_50_ values. Nonetheless, the overall rank of drug potency is the same for both assays.

**Figure 2:**
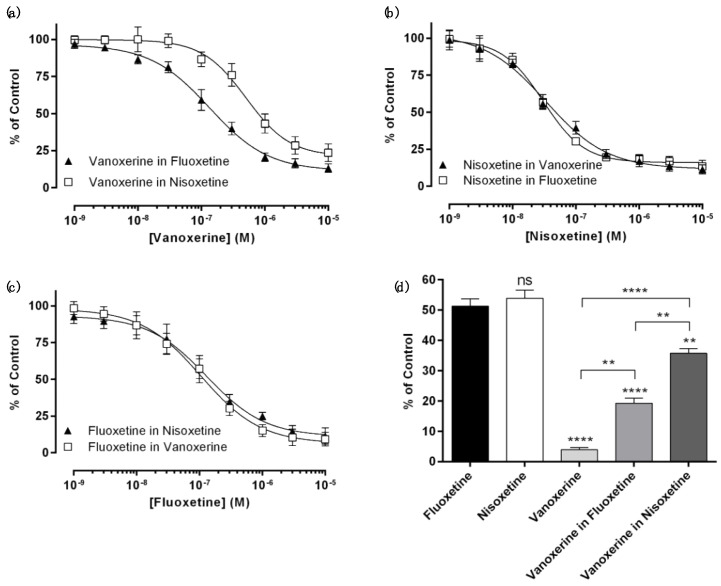
Monoamine transporter inhibition by multiple drugs: (**a**) DAT inhibition by vanoxerine with fluoxetine or nisoxetine. (**b**) NET inhibition by nisoxetine with vanoxerine or fluoxetine. (**c**) SERT inhibition by fluoxetine with nisoxetine or vanoxerine. (**d**) DAT inhibition by fluoxetine at 18.4 μM, nisoxetine at 1.15 μM, and vanoxerine at 0.3 μM in the absence and presence of fluoxetine or nisoxetine in (**a**). The transporter activities in the presence of nisoxetine in (**a**), fluoxetine in (**b**), vanoxerine in (**c**), and DMSO in (**d**) were used as controls of (**a**–**d**), respectively. ** *p* < 0.01 and **** *p* < 0.0001. ns is not significant.

The effects of multiple drugs on monoamine transporter inhibition were then examined. Specifically, monoamine transporter inhibition by their selective inhibitor (DAT inhibition by vanoxerine, NET inhibition by nisoxetine, and SERT by fluoxetine) was measured in the presence of other drugs at their IC_50_ values. DAT inhibition by vanoxerine was carried out in the presence of 1.15 μM nisoxetine or 18.4 μM fluoxetine. Interestingly, vanoxerine inhibited DAT less potently in the presence of nisoxetine or fluoxetine (Figure 2 and Table 2). Quantitatively, fluoxetine resulted in a 1.6-fold increased IC_50_ value of vanoxerine for DAT, and nisoxetine showed an even more dramatic increase in the IC_50_ values by six folds. For NET or SERT inhibition, however, multiple-drug treatments did not affect drug potency (Figure 2 and Table 2).

It is noteworthy that the minimum percent activity (%A*_min_*) values of DAT by vanoxerine seem to also be affected by co-treatment with other drugs (Table 2). When vanoxerine was the only treatment, DAT was completely inhibited; the remaining DAT activity was ~0%. In the presence of fluoxetine, however, vanoxerine did not complete DAT inhibition, with ~10% residual activity. Nisoxetine produced a more severe effect, with a %A*_min_* value of 22. Because nisoxetine or fluoxetine alone did not completely inhibit DAT (a %A*_min_* value of nine for both drugs, Table 1), the 10% residual DAT activity in the presence of vanoxerine and fluoxetine might have been solely due to fluoxetine. However, the combination of vanoxerine and nisoxetine caused 22% residual DAT activity, which is twice as high as that achieved via the simple addition of the %A*_min_* values of each drug. Along with the six-fold increased IC_50_ value of vanoxerine in the presence of nisoxetine, the 22% residual DAT activity suggests antagonistic effects of vanoxerine and nisoxetine.

As addressed in the Introduction section, the force driving dopamine uptake by DAT is the concentration gradients of Na^+^ and Cl^−^. Consequently, dopamine uptake does not occur when either Na^+^ or Cl^−^ is absent. Interestingly, however, other ions act like a DAT inhibitor [9], suggesting that their competition with Na^+^ or Cl^−^ binding reduces the force driving dopamine transport. Thus, ions other than Na^+^ or Cl^−^ could exert different effects on DAT inhibition by vanoxerine, possibly through affecting Na^+^/Cl^−^ binding as well as substrate/inhibitor binding.

### 3.2. Effects of Monovalent Ions on DAT Inhibition by Vanoxerine

**Figure 3:**
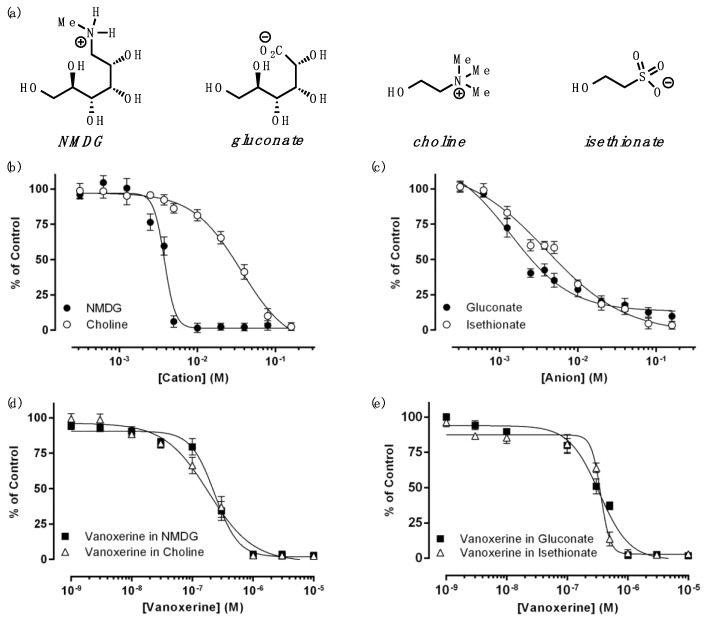
DAT inhibition by monovalent ions and vanoxerine with monovalent ions: (**a**) The structures of monovalent ions. (**b**) DAT inhibition by NMDG and choline. (**c**) DAT inhibition by gluconate and isethionate. (**d**) DAT inhibition by vanoxerine with NMDG or choline. (**e**) DAT inhibition by vanoxerine with gluconate or isethionate. Transporter activities in the absence of monovalent ions in (**b**,**c**), and choline in (**d**) and gluconate (**e**) were used as controls of (**b**–**e**), respectively.

To further investigate the antagonistic effects of multiple inhibitors, we examined ion dependency in DAT inhibition by vanoxerine. First, various ions, different in size and charge, were tested for DAT inhibition (Figure 3a). Both monovalent cations (NMDG and choline) and anions (gluconate and isethionate) inhibited substrate uptake by DAT in a concentration-dependent manner, with IC_50_ values ranging from 1 to 37 mM, exhibiting complex inhibitory effects associated with their size and charge (Figure 3b,c and Table 3). Overall, anions seemed to be more potent than cations, and big sugar ions (gluconate and NMDG) appeared to be more potent than small ions (isethionate and choline) for DAT inhibition. In particular, choline was the least potent inhibitor of DAT, with a potency approximately 10-fold lower than that of the other ions tested in this work. Even though choline exists endogenously, the choline levels in the brain have been reported to range between 10 and 100 μM [10,11], which an order of magnitude lower than the IC_50_ value of choline for DAT inhibition (37 mM). Thus, choline does not appear to have any significant effects on DAT in vivo. It is also worth noting that NMDG produces a stiff sigmoidal curve with an apparent Hill coefficient (*h*) of 11, while other ions have *h* values of 1. This suggests that DAT has multiple binding sites for NMDG.

Then, DAT inhibition by vanoxerine was examined in the presence of monovalent cations, NMDG or choline, or in the presence of monovalent anions, gluconate or isethionate (Figure 3d,e and Table 4). For these experiments, the IC_50_ values of each monovalent ion were used, i.e., 3.7 mM NMDG, 36.6 mM choline, 1.4 mM gluconate, or 3.3 mM isethionate. Like the multiple drug effects described above, monovalent ions reduced vanoxerine potency by two to three folds. In particular, isethionate appeared to alter DAT inhibition by vanoxerine the most, increasing the IC_50_ value by three folds. This might be because isethionate contains a chemical moiety with a sulfur atom, as observed in some atypical DAT inhibitors. This result is consistent with that of a previous report that isethionate reduced the binding of a DAT inhibitor, mazindol, by more than 50% [12]. Choline and NMDG have been also reported to interfere with DAT inhibitor binding [13,14,15,16]. Altogether, anions seemed to affect vanoxerine inhibition more than cations, although the difference among the ions was not significant.

### 3.3. Structural Insights of DAT Inhibition

Molecular docking studies with the X-ray crystal structures of LeuT have revealed that inhibitors bind either to the central binding site (S1 site) that overlaps the substrate (leucine) or to the secondary binding site (S2 site) in the extracellular vestibule [17,18,19]. DAT homology models built based on the LeuT structures also suggest two different binding sites (S1 and S2 sites) for inhibitors [20,21]. The majority of inhibitor binding occurs at the S1 site, where the charged amine group of the inhibitor interacts with the carboxyl group of Asp98. The importance of the interactions between the amine group of the substrate/inhibitor and Asp98 in DAT was also observed in the docking results for dopamine and cocaine to DAT [22].

**Figure 4:**
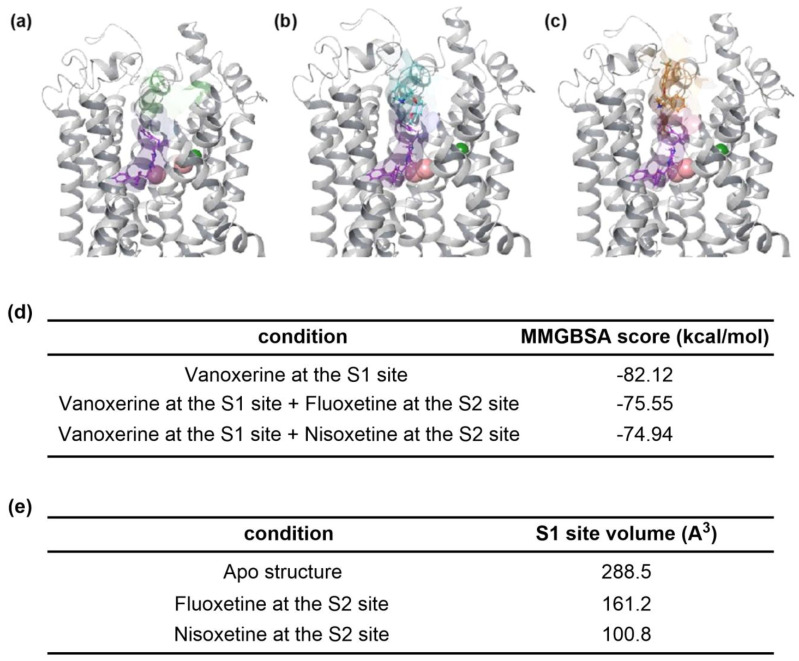
Molecular modeling analysis between homology models of hDAT and monoamine reuptake inhibitors. Representative complexes of hDAT and vanoxerine (purple) (**a**), vanoxerine with fluoxetine (cyan) (**b**), and vanoxerine with nisoxetine (orange) (**c**). (**d**) MMGBSA scores derived from the complexes. (**e**) S1 binding site volume calculated from 300 ns MD simulations. Vanoxerine is docked to the S1 site of the hDAT structure, and fluoxetine and nisoxetine are docked to the S2 site. The green and red spheres represent chloride ions and sodium ions, respectively.

In order to describe this antagonistic effect at atomic scale using the three-dimensional structure of hDAT, a molecular modeling study was conducted with the hDAT model. The S1 site is located at the center of the transport pathway of hDAT, and the S2 site is reported to be at the entrance of the transport pathway. Docking simulations were performed on sites S1 and S2, respectively, to predict the binding sites of nisoxetine and fluoxetine in the presence of vanoxerine. Based on the docking results, it was predicted that vanoxerine preferentially binds to the S1 site of hDAT, but nisoxetine and fluoxetine are expected to bind to the S2 site in the presence of vanoxerine (Figure 4a–c).

As a result of calculating the average binding energies of vanoxerine by analyzing the simulation trajectories, the binding energy of vanoxerine to hDAT is weakened when nisoxetine or fluoxetine is bound to the S2 site (Figure 4d). This energy change indicates that the binding of nisoxetine or fluoxetine at the S2 site might induce a conformational change of the S1 site. To elucidate these structural changes, additional MD simulations were performed, and the structural changes were compared. The Apo-hDAT structure has 288.5 A3 average binding site volumes for site S1 (Figure 4e). However, the volumes of the S1 site significantly decrease when nisoxetine or fluoxetine is bound at the S2 site. We think that antagonistic effects were observed due to the binding of nisoxetine or fluoxetine at the S2 site, which reduced the space for binding at the S1 site. As a result, the binding energy of vanoxerine binding to the S1 site was weakened in the presence of nisoxetine or fluoxetine.

## 4. Conclusions

In the present work, we observed the antagonistic effects of multiple drugs on DAT inhibition by monoamine transporter selective inhibitors. It was also observed in a different class of inhibitors, monovalent ions. Also, molecular modeling studies were performed to understand the antagonistic effects of vanoxerine with nisoxetine or fluoxetine. This persistent antagonistic effect on DAT inhibition observed here suggests that a combinatorial therapeutic strategy using monoamine reuptake inhibitors should be carefully considered. Since this study has a limitation in that multiple drugs were only tested on the cells stably overexpressing monoamine transporters, it is necessary to conduct additional verification on primary neuronal cells in the future. Moreover, if the arguments proposed in this study can be verified through in vivo animal models mimicking DAT-related diseases such as depression and schizophrenia, they can be extended to the individual level.

## Figures and Tables

**Table 1:** Drug potencies of monoamine transporters.

		Vanoxerine	Nisoxetine	Fluoxetine
DAT	IC_50_ (μM)	0.09 ± 0.01	1.15 ± 0.17	18.4 ± 2.7
	%A*_min_*	0.0 ± 0.8	9.2 ± 2.4	9.4 ± 2.8
NET	IC_50_ (μM)	1.46 ± 0.13	0.019 ± 0.001	4.41 ± 0.33
SERT	IC_50_ (μM)	3.84 ± 0.22	0.70 ± 0.07	0.13 ± 0.03

The half-maximum inhibition concentration (IC_50_) values and the minimum percent activity (%A*_min_*) values were obtained by fitting the dose–response curves to a Hill equation, presented as the mean ± SEM. Independent experiments were performed (*n* = 4–5).

**Table 2:** Drug potencies for monoamine transporters in the presence of other drugs.

DAT		Vanoxerine	Vanoxerine in Fluoxetine	Vanoxerine in Nisoxetine
	IC_50_ (μM)	0.09 ± 0.01	0.14 ± 0.03	0.55 ± 0.09
	%A*_min_*	0.0 ± 0.8	10.5 ± 3.5	21.7 ± 5.6
	Significance		*	****
NET		Nisoxetine	Nisoxetine in Vanoxerine	Nisoxetine in Fluoxetine
	IC_50_ (μM)	0.019 ± 0.001	0.032 ± 0.002	0.030 ± 0.02
	Significance		NS	NS
SERT		Fluoxetine	Fluoxetine in Nisoxetine	Fluoxetine in Vanoxerine
	IC_50_ (μM)	0.13 ± 0.03	0.13 ± 0.04	0.11 ± 0.03
	Significance		NS	NS

The half-maximum inhibition concentration (IC_50_) values and the minimum percent activity (%A*_min_*) values were obtained by fitting the dose–response curves to a Hill equation, and are presented as the mean ± SEM. The significance of observed differences was evaluated using Kruskal–Wallis and post hoc Dunn’s tests. A *p* value of <0.05 was considered statistically significant. * *p* < 0.05 and **** *p* < 0.0001. NS is not significant. Independent experiments were performed (*n* = 3–5).

**Table 3:** DAT inhibition by monovalent ions.

	Cation		Anion	
	NMDG	Choline	Gluconate	Isethionate
IC_50_ (mM)	3.7 ± 0.2	36.6 ± 2.0	1.4 ± 0.2	3.3 ± 0.6
*h*	10.6 ± 3.8	1.4 ± 0.2	1.2 ± 0.3	0.8 ± 0.2

The half-maximum inhibition concentration (IC_50_) values and the Hill coefficient (*h*) values were obtained by fitting the dose–response curves to a Hill equation and are presented as the mean ± SEM. Independent experiments were performed (*n* = 6).

**Table 4:** DAT inhibition by vanoxerine in the presence of monovalent ions.

	Control	Cation		Anion	
		NMDG	Choline	Gluconate	Isethionate
IC_50_ (μM)	0.09 ± 0.01	0.17 ± 0.01	0.14 ± 0.01	0.20 ± 0.02	0.23 ± 0.01
Significance		***	**	**	****
		NS		NS	

The half-maximum inhibition concentration (IC_50_) values were obtained by fitting the dose–response curves to a Hill equation and are presented as the mean ± SEM. The significance of observed differences was evaluated by Kruskal–Wallis and post hoc Dunn’s tests. A *p* value of <0.05 was considered statistically significant. ** *p* < 0.01, *** *p* < 0.001, and **** *p* < 0.0001. NS is not significant. Independent experiments were performed (*n* = 4).

## Data Availability

Not applicable.

## Supplementary Materials

The following supporting information can be downloaded at: https://www.mdpi.com/article/10.3390/biomedicines11102846/s1, Figure S1: Time-dependent substrate uptake and concentration-dependent inhibition; Figure S2: Experimental vs. literature drug potencies for monoamine transporters; Table S1: Experimental vs. literature drug potencies for monoamine transporters in substrate uptake assays.
