# Supplementary material for: Implications for Combination Therapy of Selective Monoamine Reuptake Inhibitors on Dopamine Transporters
Source: Biomedicines. 2023 Oct 20;11(10):2846. doi: 10.3390/biomedicines11102846 (PMC10604324; doi:10.3390/biomedicines11102846)
Supplement: Supplementary file 1 [file biomedicines-11-02846-s001.zip › biomedicines-2622592-supplementary.pdf]

Figure S1. Time-dependent substrate uptake and concentration-dependent inhibition. The time courses of substrate uptake by HEK293 cells expressing DAT (A), NET (B), and SERT (C) are presented as a ratio of the measured fluorescent intensity to the initial fluorescent intensity (F_t/F_0). A selective drug inhibited substrate uptake by each monoamine transporter in a concentration-dependent fashion: DAT inhibition by vanoxerine (A), NET inhibition by nisoxetine (B), and SERT inhibition by fluoxetine (C). The change in intracellular substrate concentration was monitored as the change in fluorescent intensity for 30 min. Error bars represent s.e.m.

A.

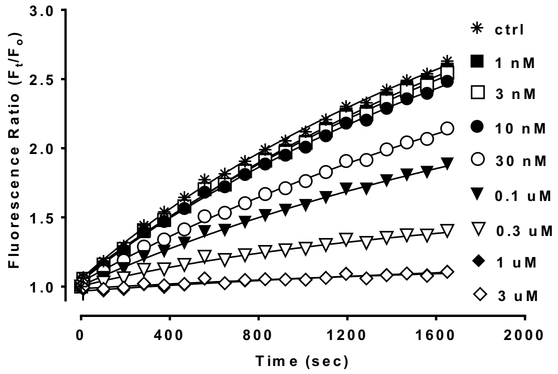

B.

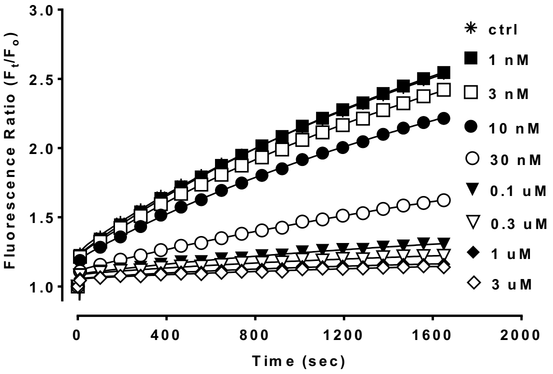

C.

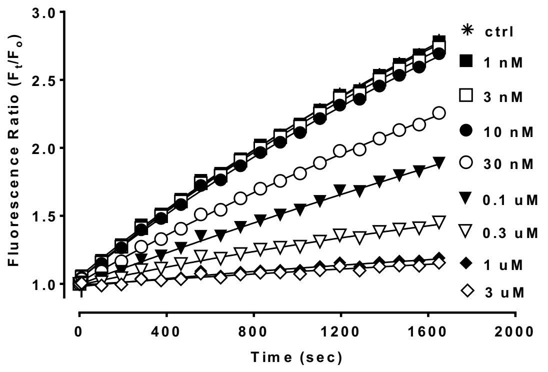

Figure S2. Experimental *vs.* literature drug potencies for monoamine transporters. Experimental IC_{50} values are plotted against literatures IC_{50} values for all monoamine transporters. Experimentally obtained data are systematically bigger than the data from the literatures. A dotted linear line was added to make the systematic difference easily noticed.

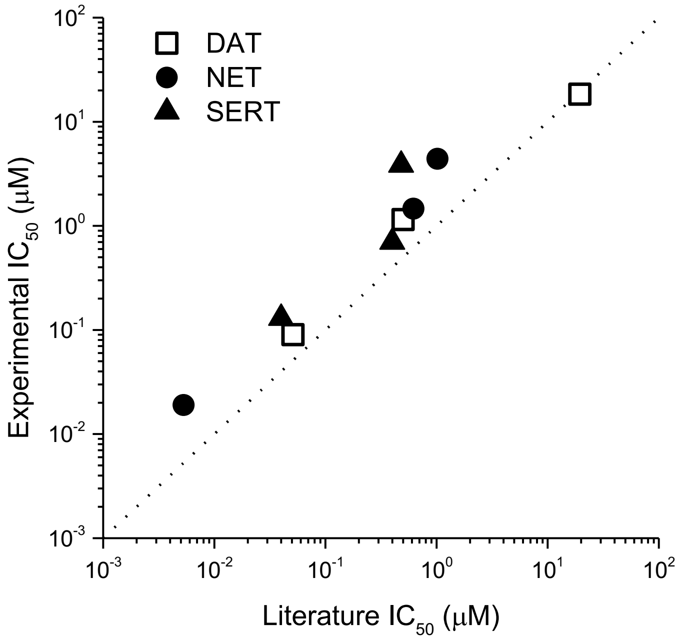

Table S1. Experimental *vs.* literature drug potencies for monoamine transporters in substrate uptake assays.

		Vanoxerine	Nisoxetine	Fluoxetine
DAT	Experimental IC_{50} (μM)	0.09	1.15	18.4
	Literature IC_{50} (μM)	0.051 [a]	0.50 [b]	19.5 [b]
	Ratio (Exp. IC_{50}/Lit. IC_{50})	1.8	2.3	0.9
NET	Experimental IC_{50} (μM)	1.46	0.019	4.41
	Literature IC_{50} (μM)	0.62 [a]	0.0053 [b]	1.02 [b]
	Ratio (Exp. IC_{50}/Lit. IC_{50})	2.4	3.6	4.3
SERT	Experimental IC_{50} (μM)	3.84	0.70	0.13
	Literature IC_{50} (μM)	0.48 [a]	0.40 [b]	0.04 [c]
	Ratio (Exp. IC_{50}/Lit. IC_{50})	8.0	1.8	3.3

Exp. IC_{50} values are experimentally obtained values in this work, and Lit. IC_{50} values are previously reported values in literatures. [a] M.J. Hansard, *et al.*, J Pharmacol Exp Ther 303 (2002) 952-958. [b] A.J. Eshleman, *et al.*, J Pharmacol Exp Ther 289 (1999) 877-885. [c] J.V. Frnka, *et al.*, J Pharmacol Exp Ther 256 (1991) 734-740.
